# Identification of novel indole derivatives acting as inhibitors of the Keap1–Nrf2 interaction

**DOI:** 10.1080/14756366.2019.1623209

**Published:** 2019-06-09

**Authors:** Barbara Cosimelli, Giovanni Greco, Sonia Laneri, Ettore Novellino, Antonia Sacchi, Giorgio Amendola, Sandro Cosconati, Roberta Bortolozzi, Giampietro Viola

**Affiliations:** a Dipartimento di Farmacia, Università di Napoli “Federico II”, Naples, Italy;; b Dipartimento di Scienze e Tecnologie Ambientali Biologiche e Farmaceutiche, Università degli Studi della Campania “Luigi Vanvitelli”, Caserta, Italy;; c Dipartimento di Salute della Donna e del Bambino, Università di Padova, Padua, Italy

**Keywords:** Indole derivatives, Keap1–Nrf2 interaction, Keap1–Nrf2-ARE system, oxidative stress, antioxidant response element

## Abstract

Nine indole derivatives (**9a-i**) were tested as potential inhibitors of the Keap1–Nrf2 interaction. This class of compounds increases the intracellular levels of the transcription factor Nrf2 and the consequent expression of enzymes encoded by genes containing the antioxidant response element (ARE). In the ARE-luciferase reporter assay only **9e**-**g** revealed to be remarkably more active than *t*-butylhydroxyquinone (*t*-BHQ), with **9g** standing out as the best performing compound. While **9e** and **9f** are weak acids, **9g** is an ampholyte prevailing as a zwitterion in neutral aqueous solutions. The ability of **9e-g** to significantly increase levels of Nrf2, NADPH:quinone oxidoreductase 1, and transketolase (TKT) gave further support to the hypothesis that these compounds act as inhibitors of the Keap1–Nrf2 interaction. Docking simulations allowed us to elucidate the nature of the putative interactions between **9g** and Keap1.

## GRAPHICAL ABSTRACT

**Figure:**
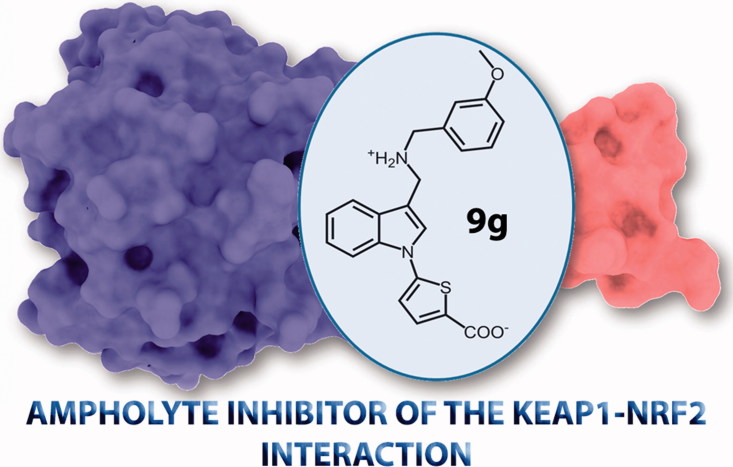

## Introduction

The interaction between Kelch-like ECH-associated protein 1 (Keap1)^1^ and the Nuclear Factor Erythroid 2-Related Factor 2 (Nrf2)^2^ plays a crucial role in the homeostasis of cellular oxidative stress^3^. Under physiological conditions, the activity of Nrf2 as a transcription factor is negatively regulated by Keap1 through proteasomal degradation mechanisms^4^. An increase of the intracellular levels of reactive oxygen species (ROS) interferes with the stability of the Keap1–Nrf2 complex – situated in the cytoplasm – by oxidation of cysteine residues located in a specific domain of Keap1. Disruption of the stability of the Keap1–Nrf2 complex triggers the release of Nrf2, allowing this protein to reach the nucleus where it behaves as a transcriptional activator of genes that contain the enhancer sequence antioxidant response element (ARE)^5^. As a result, several enzymes, such as NADPH:quinone oxidoreductase 1 (NQO1), heme oxygenase-1 (HO-1), glutathione S-transferase (GST), superoxide dismutase (SOD), catalase (CAT), and transketolase (TKT) are overexpressed^6–8^. These antioxidant enzymes reduce oxidative stress and, consequently, limit cellular damages. Thus, the Keap1–Nrf2-ARE system plays a pivotal role in cellular metabolism and redox balance. For the above reasons, inhibitors of the Keap1–Nrf2 interaction are currently being investigated as potential drugs to treat diseases involving chronic oxidative stress, such as diabetes, cancer and neurodegenerative disorders^9^.

The first non-peptide small molecules disrupting the Keap1–Nrf2 interaction by binding to Keap1 with micromolar affinities were reported by Hu et al.^10^ and Marcotte et al.^11^ using high throughput screening methods. This latter group also solved the 3D structures of two ligand-Keap1 complexes by X-ray crystallography^11^. The above quoted works gave impetus to additional X-ray diffraction studies and researches aimed at identifying novel ligands of Keap1^12^.

**Chart 1.**
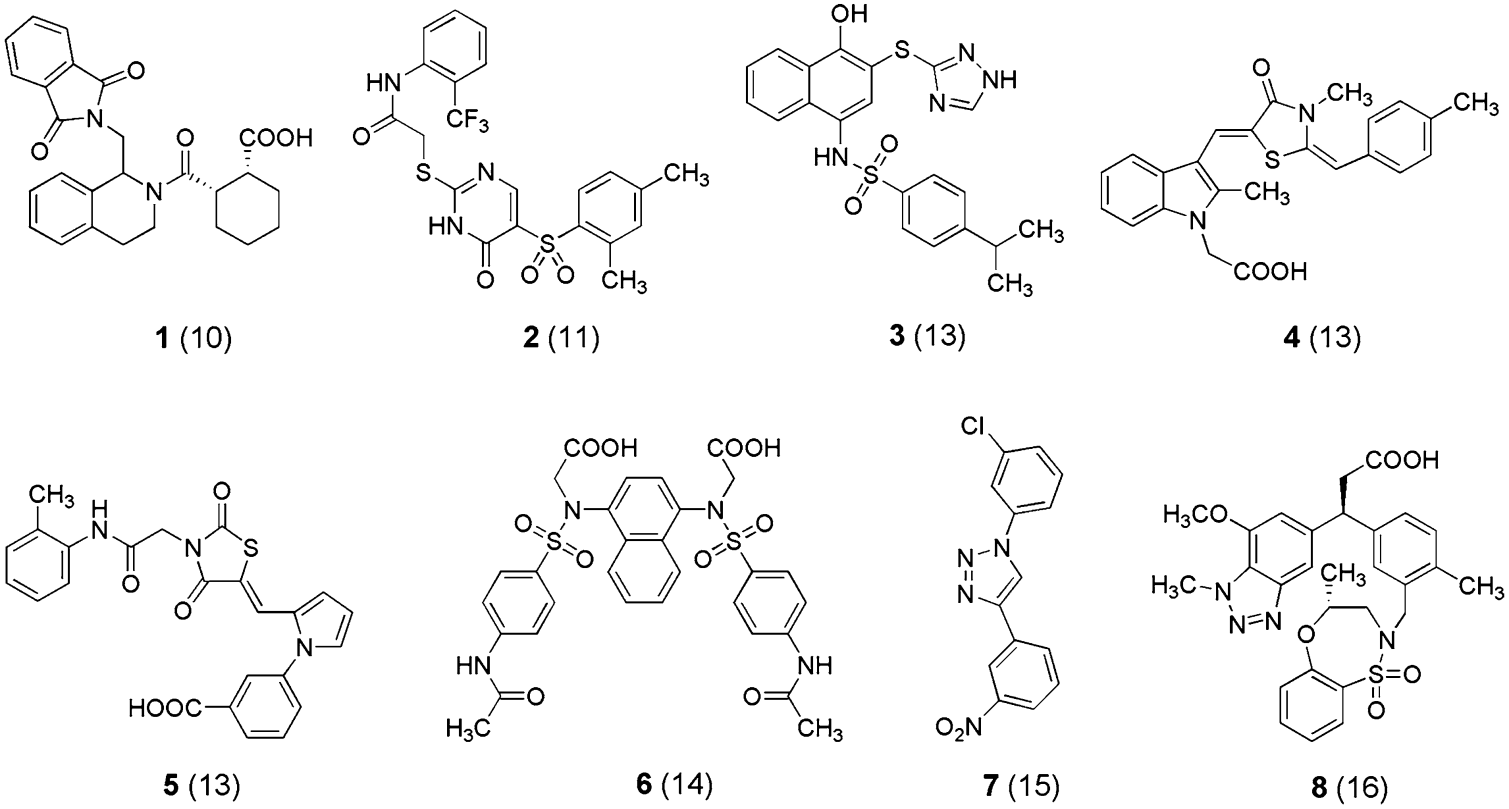
Structures of representative inhibitors of the Keap1–Nrf2 interaction. For each compound a reference is reported in parentheses. Compounds **6** and **8** exhibit nanomolar affinities for Keap1, whereas the remaining compounds bind to Keap1 with micromolar affinities.

According to Jiang et al.^12^, the Keap1 binding cavity hosting inhibitors of the Keap1–Nrf2 interaction can be divided into six subpockets (P1–P6). P1 and P2 contain protonated arginine residues (R483, R415, R380) which give rise to strong electrostatic interactions with electron-rich parts of ligands; specifically, salt bridges with carboxylate groups, H-bonds with nitro oxygens or azole nitrogens, cation–π contacts with aromatic rings. Additional polar as well as hydrophobic interactions are established between ligands and the complementary Keap1 binding cavity. Some inhibitors of the Keap1–Nrf2 interaction, representative of the variety of a huge number of those reported in literature^10–16^ are reported in Chart 1. They typically contain in their structures a planar or quasi-planar scaffold bearing at least one aromatic ring involved in cation–π interactions.

Our research group has been working for several years on different series of indole derivatives each of them binding with high potency and selectivity to the benzodiazepine receptor^17^, the A_2B_ adenosine receptor^18^ and the translocator protein^19^. Given the commonly ascertained utility of indole as a scaffold for drug-like molecules (see compound **4** in Chart 1), we tried to identify novel indole derivatives which could act as inhibitors of the Keap1–Nrf2 interaction with the help of molecular modelling and substructure search methods. Specifically, a few indole-bearing models were superimposed on available 3D structures of inhibitors of the Keap1–Nrf2 interaction co-crystallised with Keap1. Starting from some designed indole derivatives we identified commercially available compounds using the SciFinder^20^ substructure search routine. Examples of the above approaches are given in the Supporting Information. The present paper describes the results of our studies.

## Results

### Chemistry

**Chart 2.**
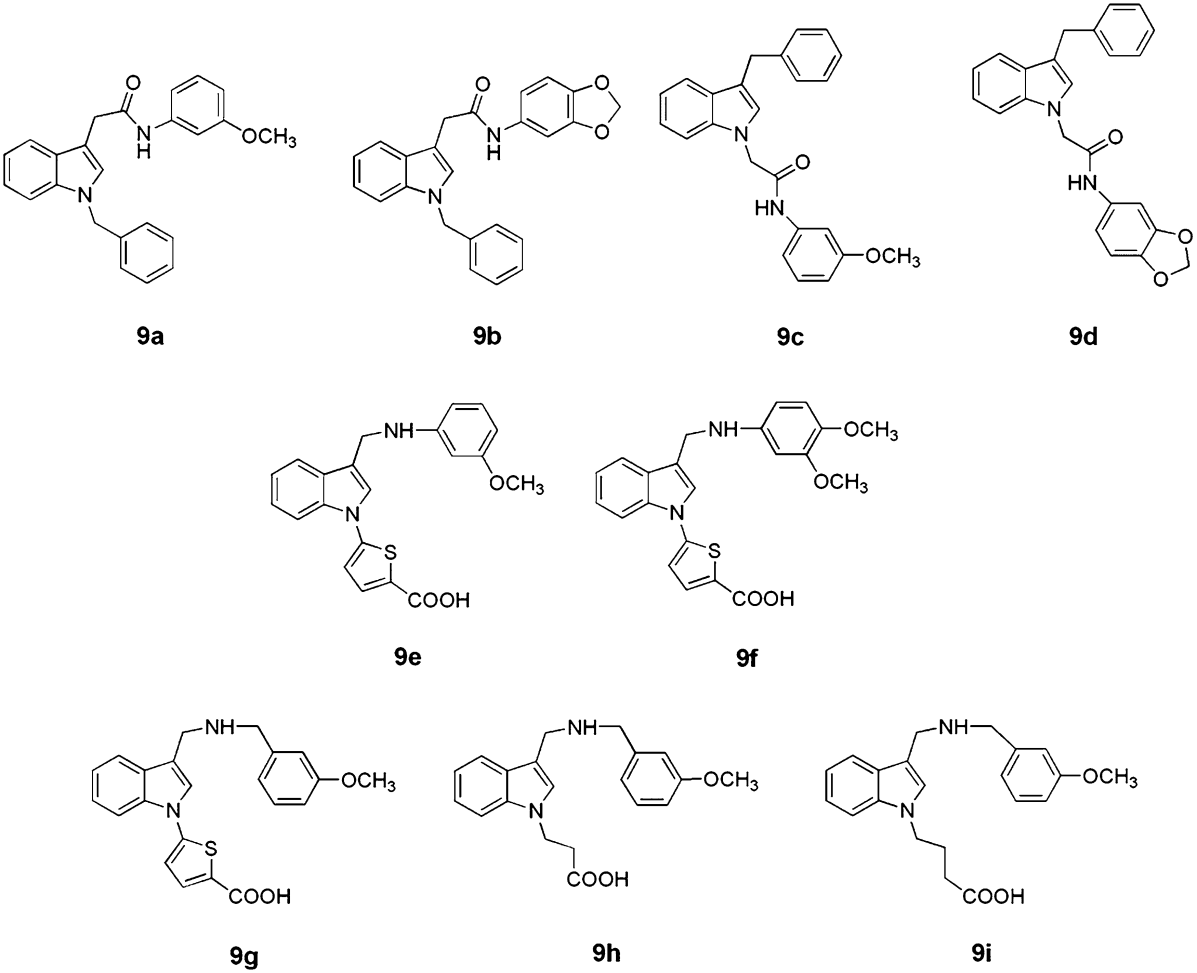
Structures of the indole derivatives selected as potential inhibitors of the Keap1–Nrf2 interaction.

Nine indole derivatives fulfilling the pharmacophoric requirements to act as inhibitors of the Keap1–Nrf2 interaction^12^ were selected for biological evaluation (**9a-i** reported in Chart 2). Compounds **9a-d** were synthesised (see Supporting Information), whereas **9e-i** were purchased from AKos (AKos GmbH, Steinen, Germany). Based on their acid-basic properties, these compounds can be divided into three groups: (a) non-ionisable (**9a-d**); (b) acidic (**9e**, **f**); ampholytic (**9g-i**). We reasoned that the presence of methoxy group(s) or a methylendioxy moiety on the benzene rings in the structures of the selected compounds might allow them to interact with Keap1 through cation–π interactions and H-bonds. The thiophene ring featured by **9e-g** confers more conformational rigidity to the above compounds and an electron-rich ring capable to establish cation–π interactions.

## Biology

The biology experiments are detailed in the Supporting Information. To evaluate the capability of the indole derivatives **9a-i** to inhibit the Keap1–Nrf2 interaction, we performed a cell-based luciferase reporter assay in which induction of ARE-driven luciferase activity is mediated by Nrf2. HeLa cells were transiently transfected with ARE-luciferase reporter plasmids and treated with all compounds at the concentration of 10 μM, except for **9g** which was tested at the concentration of 5 μM owing to its limited solubility in phosphate buffer. *t*-Butylhydroxyquinone (*t*-BHQ), one of the canonical activators of Nrf2^12^^,^^21^^,^^22^, was employed as a positive control at the concentration of 50 µM, a value which induced in our experiments the maximum luciferase activity.

**Figure 1.**
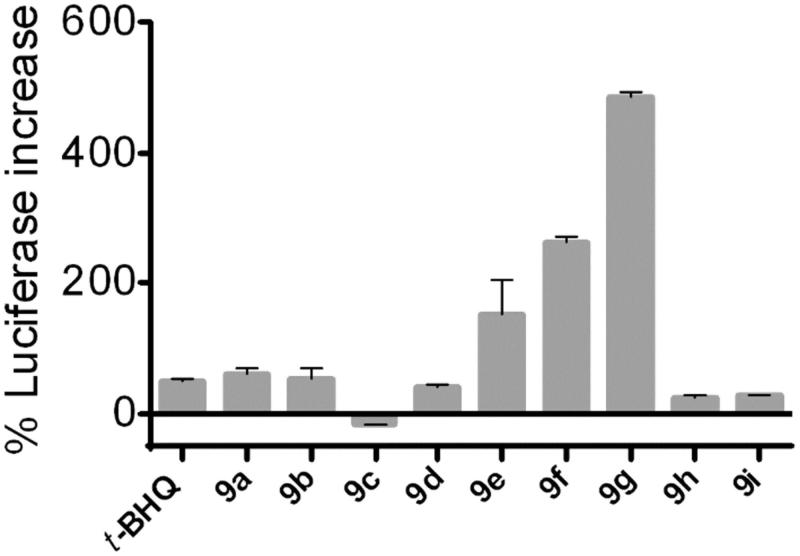
Increase of luciferase activity in HeLa cells transfected transiently with ARE-luciferase reporter plasmids after 24 h of treatment with 10 µM of the indicated compounds, except for **9g** tested at the concentration of 5 µM due to its limited solubility in phosphate buffer. Data are expressed as mean ± SEM of two independent experiments performed in duplicate.

Compounds **9e**, **9f**, and **9g** increased luciferase activity by 152, 263, and 486%, respectively (Figure 1). Compared with the activity exhibited by *t*-BHQ (48% increase), the activities of **9e-g** were higher by 3.2-, 5.5-, and respectively, 10.1-folds. The remaining compounds displayed poor activities in this assay, comparable or lower with respect to *t*-BHQ. The best performing compounds **9e-g** share a common thiophene-carboxylic moiety. Among this subset, **9e** and **9f** are acidic, whereas **9g** – by far the most active compound – is ampholytic.

**Figure 2.**
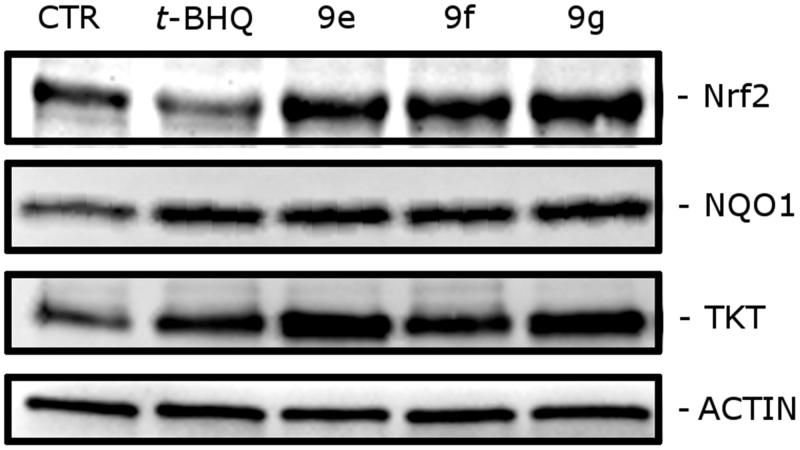
Western blot analysis of Nrf2, NQO1 and TKT after 24 h of treatment of HeLa cells with the indicated compounds. Compounds **9e** and **9f** were used at the concentration of 10 µM, whereas **9g** was used at the concentration of 5 µM due to its limited solubility in phosphate buffer. *t*-BHQ was used at the concentration of 50 µM as a positive control. To confirm equal protein loading, each membrane was stripped and reprobed with anti-β-actin antibody.

To assess the inhibition of the Keap1–Nrf2 interaction exerted by **9e-g**, we evaluated the expression of Nrf2 and of two enzymes encoded by its downstream target ARE genes, namely NQO1 and TKT. Hela cells were treated with **9e** and **9f** at the concentration of 10 µM while **9g** was employed at the concentration of 5 µM. We used *t*-BHQ as a reference compound at the concentration of 50 µM. As shown in Figure 2, expression of NQO1 and of TKT remarkably increased after treatment with **9e-g**. In Figure 2, it can be appreciated a significant increase in Nrf2 levels determined by our indole derivatives, clearly much higher compared to that produced by *t*-BHQ. The original picture of the Western blot gel is reported in the Supporting Information.

**Figure 3.**
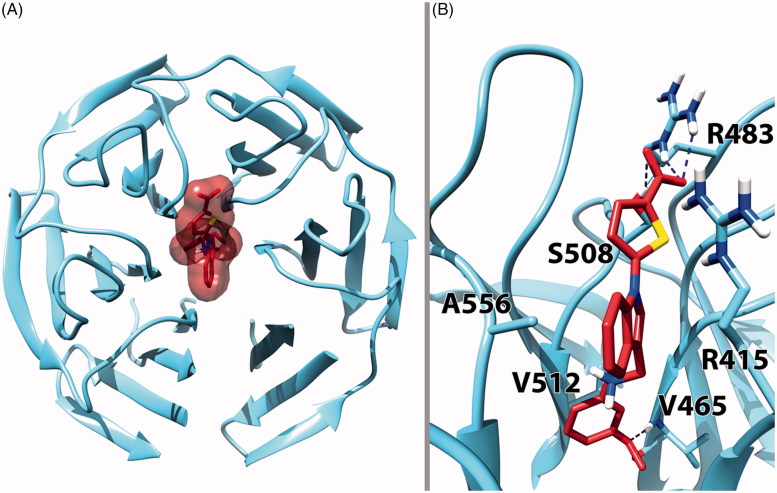
Top (A) and front view (B) of the predicted binding conformation of **9g** in complex with Keap1 extracted from the X-ray structure having PDB code 4L7B^20^. In panel A, the protein is represented as cyan ribbons while the ligand as brown sticks and transparent surface. In panel B, the protein is represented as cyan sticks and ribbons while the ligand as red sticks. H-bond interactions are evidenced with dashed blue lines.

Compounds **9e-g**, selected as the most active indole derivatives in the luciferase assay, were evaluated for their cytotoxicity after 72 h of incubation in human peripheral blood lymphocytes (PBLs) (Table S1). The compounds were tested either in quiescent and in proliferating PBLs (proliferation was induced by phytohematoagglutinin (PHA) as a mitogenic stimulus).

The obtained data indicate that the above compounds can be considered safe for human cells. In the absence of PHA, **9e**, **f** exhibited GI_50_ values greater than 100 µM, while **9g** showed GI_50_ values greater than 5 µM (higher concentrations of this compound could not be used due to its limited solubility in phosphate buffer). Compounds **9e**, **f** slightly increased their cytotoxicity in proliferating PBLs showing GI_50_ values of 61.0 μM and, respectively, 84.9 µM. Compound **9g** killed 6 and 10% of quiescent and, respectively, proliferating PBLs.

### Molecular modelling

**Figure 4.**
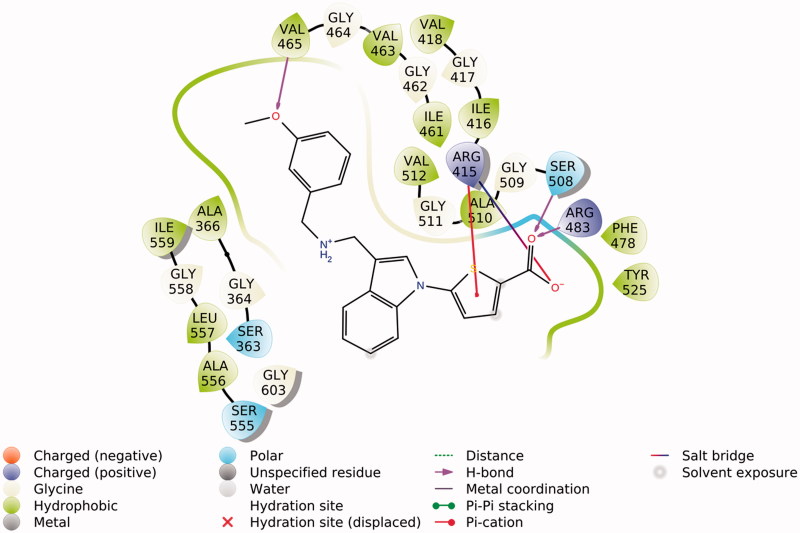
2D scheme of interaction between **9g** docked pose and Keap1 corresponding to the docking model depicted in Figure 3.

The potential interactions of **9g** with Keap1 were modelled *in silico* by docking simulations. Details of these calculations are available in the Supporting Information. Compound **9g** was treated as a zwitterion, as this species prevails to a significant extent either in neutral aqueous solutions or in the essentially polar environment of the Keap1 binding cavity. As represented in Figures 3 and 4, in our docking model the ligand occupies the central cavity of Keap1 with the carboxylate group pointing outwards and the rest of the molecule extending into the central channel of the protein. Based on the partition of the Keap1 binding cavity proposed by Jiang et al.^12^ into subpockets P1–P6, **9g** seemingly occupies mainly P1 and part of P2 and P3, taking into account that arginine 415 is located at the border of P1 and P2.

Several strong electrostatic ligand–protein interactions take place: the carboxylate of **9g** forms a salt bridge with the arginine 483 side chain and a charge-reinforced H-bond with the P1 serine 508 side chain; the thiophene ring of the ligand establishes a cation–π interaction with the arginine 415 side chain (subpockets P1 and P2). Weaker hydrophobic interactions contribute to the ligand-protein affinity through favorable contacts between the indole nucleus of the ligand with the P3 alanine 556 methyl group and the arginine 415 dimethylene fragment.

The (*m*-methoxy)benzylaminomethyl substituent, in its protonated state, points deep down into the central Keap1 channel where it establishes hydrophobic interactions with valine 512 and leucine 472 side chains and a H-bond between the *m*-methoxy oxygen and the leucine 472 backbone NH.

The results of our theoretical calculations suggest that the thiophene ring featured by **9e**-**g**, engaged in a strong cation–π interaction, is responsible for their considerable activities as inducers of antioxidant enzymes. This hypothesis is consistent with the much lower activities exhibited by **9h** and **9i** in which a dimethylene and, respectively, a trimethylene chain – in place of the thiophene moiety – bear a carboxyl group. However, the entropic advantage offered by the thiophene ring in reducing the conformational freedom of **9e**-**g** with respect to **9h**, **i** cannot be ruled out.

## Discussion

The results of the biological experiments and the consistency of our model of the **9g**-Keap1 complex with SARs suggest that **9e-g** act as inhibitors of the Keap1–Nrf2 interaction. These three compounds share a thiophene-carboxylate moiety which gives rise to putative strong electrostatic interactions with arginine 483 and serine 508 of Keap1 and, additionally, limits conformational freedom.

Acidic inhibitors of the Keap1–Nrf2 interaction bearing carboxylic groups exist in aqueous neutral solution mainly as anionic species. This have been considered an obstacle to translocation into cells^15^^,^^23^. To circumvent such a problem, bioisosteric replacements of a carboxylic group with a tetrazole ring^24^ or a nitro group^16^ have been attempted, yielding compounds which retained high affinity for Keap1 and exhibited improved activity in cell-based experiments. Compound **9g** stands out as the most active indole derivatives among those investigated. To our knowledge, **9g** represents the first inhibitor of the Keap1–Nrf2 interaction with ampholytic properties.

Several physicochemical and pharmacokinetic properties of **9a-i** were calculated using the Maestro QikProp tool^25^ (Table S1). The drug-likeness of the compounds is indeed confirmed by these data that show a negligible number of Lipinski Rule of 5 and Jorgensen Rule of 3 violations, good oral absorption and a prevalently lipophilic profile for each of them.

The data reported in the present paper, together with the docking model of **9g**-Keap1 complex, will be exploited for continuing the design and the synthesis of novel indole derivatives as inhibitors of the Keap1–Nrf2 interaction.

## Supplementary Material

Supplemental Material
