# Supplementary material for: Identification of novel indole derivatives acting as inhibitors of the Keap1–Nrf2 interaction
Source: J Enzyme Inhib Med Chem. 2019 Jun 9;34(1):1152–7. doi: 10.1080/14756366.2019.1623209 (PMC6567006; doi:10.1080/14756366.2019.1623209)
Supplement: Supplemental Material [file IENZ_A_1623209_SM6901.pdf]

Supporting Information

Identification of novel indole derivatives acting as inhibitors of the Keap1-Nrf2 interaction

Barbara Cosimelli,[a] Giovanni Greco,*[a] Sonia Laneri,[a] Ettore Novellino,[a] Antonia Sacchi,[a] Giorgio Amendola, [b] Sandro Cosconati,[b] Roberta Bortolozzi,[c] Giampietro Viola,[c]

[a] Dipartimento di Farmacia, Università di Napoli “Federico II”, Via Domenico Montesano 49, 80131, Napoli, Italy

[b] Dipartimento di Scienze e Tecnologie Ambientali Biologiche e Farmaceutiche, Università degli Studi della Campania “Luigi Vanvitelli”, Via Antonio Vivaldi 43, 81100, Caserta, Italy

[c] Dipartimento di Salute della Donna e del Bambino – SDB, Università di Padova, Via Nicolò Giustiniani 3, 35128 Padova, Italy

* **Corresponding author**. giovanni.greco@unina.it. Phone: +039 081678645. Fax: +39 081 678107.

Table of contents:

Materials and methods

- Chemistry S2
- Biology S5
- Computational chemistry S6

References S10

Materials and methods

Chemistry

Compounds **9e** (5-(3-{[(3-methoxyphenyl)amino]methyl}-1*H*-indol-1-yl)thiophene-2-carboxylic acid), **9f** (5-(3-{[(3,4-dimethoxyphenyl)amino]methyl}-1*H*-indol-1-yl)thiophene-2-carboxylic acid), **9g** (5-(3-{[(3-methoxybenzyl)amino]methyl}-1*H*-indol-1-yl)thiophene-2-carboxylic acid), **9h** (3-(3-{[(3-methoxybenzyl)amino]methyl}-1*H*-indol-1-yl)propanoic acid) and **9i** (4-(3-{[(3-methoxybenzyl)amino]methyl}-1*H*-indol-1-yl)butanoic acid) were purchased by the AKos company (AKos GmbH - Austr.26 - 79585 Steinen – Germany).

Compounds **10**,[1] **13**,[2] and **14**,[3] were obtained as previously reported. Evaporation was performed *in vacuo* (rotary evaporator). Analytical TLC was carried out on Merck 0.2 mm precoated silica gel aluminum sheets (60 F-254). Silica gel 60 (230-400 mesh) was used for column flash-chromatography. Melting points were determined using a Büchi apparatus B 540 and are uncorrected. Routine nuclear magnetic resonance spectra were recorded on a Varian Mercury$_{400}$ spectrometer operating at 400 MHz for the proton and 100 MHz for the carbon in DMSO-d_6 solution. Purity of the compounds was determined, using a Shimadzu LC-20AD SP liquid chromatograph equipped with a DDA Detector (λ = 254 nm) using a column C18 (250 mm x 4.6 mm, 5 μm, Shim-pack)). The mobile phase, delivered at isocratic flow, consisted of acetonitrile (60%) and water (40%) and a flow rate of 1.0 mL/min. All the compounds showed percent purity values ≥ 95%. Mass spectrometry data were collected on a hybrid linear ion trap LTQ Orbitrap XL™ Fourier Transform MS (FTMS) equipped with an ESI ION MAX™ source (Thermo-Fisher; San Josè, CA, USA). Elemental formulae were calculated on the mono-isotopic peak of the ion cluster through Thermo Xcalibur software (v2.2 SP1.48 Thermo Fisher San Josè, CA, USA) at 5 ppm mass tolerance.

Syntheses of compounds **9a** and **9b**

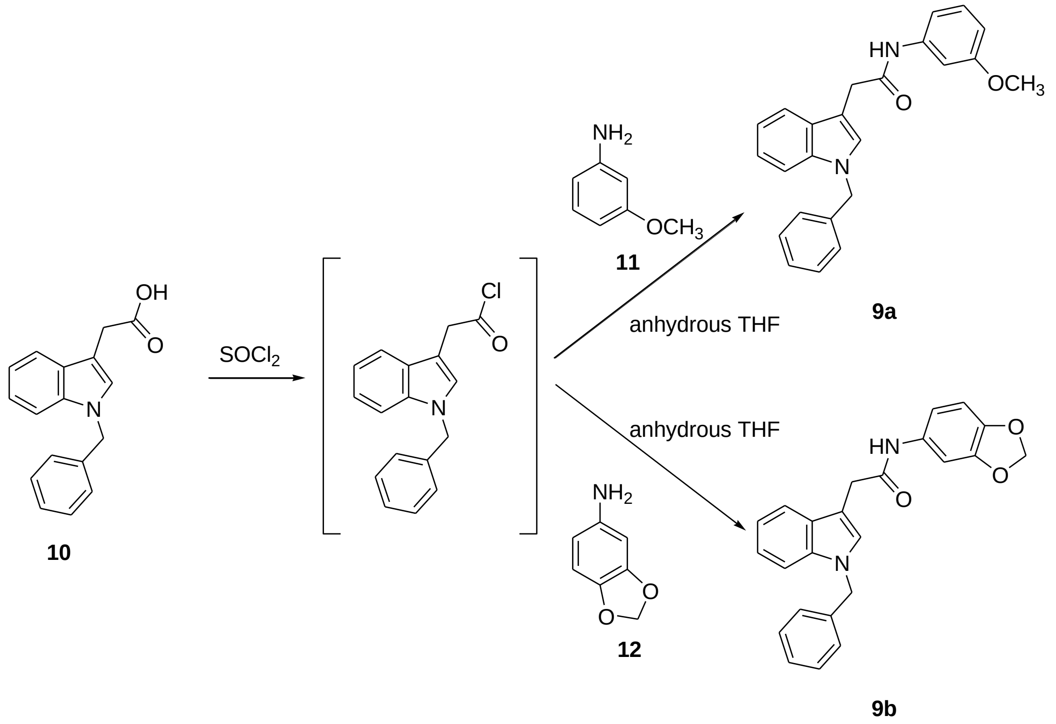

*2-(1-Benzyl-1*H*-indol-3-yl)-*N*-(3-methoxyphenyl)acetamide* **(9a)**. Thionyl chloride (0.16 mL; 2.2 mmol) was added dropwise at 0 °C to a well-stirred mixture of the 2-(1-benzyl-1*H*-indol-3-yl)acetic acid (**10**) (0.47g; 1.8 mmol) in anhydrous THF (15 mL). The mixture was maintained at 30 °C for 4 h (TLC analysis; ethyl acetate:petroleum ether = 1:1 v/v as eluent). A solution of 3-methoxyaniline (**11**) (0.4 mL; 3.5 mmol) in anhydrous THF (4 mL) was added dropwise and the mixture was stirred at room temperature for 2 h (TLC analysis; ethyl acetate:petroleum ether = 1:2 v/v as eluent). The reaction mixture was added of deionized water (5 mL) and washed with a saturated solution of $NaHCO_3$ (9 mL), then CH_2Cl_2 (15 mL) was added. The organic layer was separated and the aqueous layer was extracted with CH_2Cl_2 (3x15 mL). The collected organic phases were dried on Na_2SO_4. Removal of the solvent left a dense reddish oil which was treated with cold diethyl ether to give a gray solid. Finally the desired compound was obtained after chromatographic separation (ethyl acetate:petroleum ether = 1:2 v/v as eluent). Yield 50%; m.p. 114.5-115.8 °C (white leaflets from ethyl acetate - petroleum ether). ^{1}H NMR (DMSO-d$_6$, 400 MHz): δ 10.11 (bs, 1H, NH); 7.61 (d, *J*=7.8 Hz, 1H, H-4); 7.42 (d, *J*=7.2 Hz, 1H, H-7); 7.39 (s, 1H, H-2); 7.33-7.06 (m, 9H, H-6, Ph, H-2', H-5', H-6'); 7.01 (t, *J*=7.6 Hz, 1H, H-5); 6.60 (d, *J*=7.8 Hz, 1H, H-4'); 5.38 (s, 2H, NCH_2); 3.73 (s, 2H, CH_2CO); 3.71 (s, 3H, Me). ^{13}C NMR (DMSO-d$_6$, 100 MHz): δ 169.5; 159.5; 140.5; 138.3; 135.9; 129.4; 128.5; 127.8; 127.5; 127.3; 127.1; 121.2; 119.0; 118.7; 111.3; 110.0; 108.5; 108.4; 104.8; 54.9; 48.9; 33.6. ESI: *m/z* 371 (M + 1). HRMS: *m/z* calcd for $C_{24}H_{23}N_2O_2$: 370.1681; found: 371.1748.

N-*1,3-Benzodioxol-5-yl-2-(1-benzyl-1*H*-indol-3-yl)acetamide* **(9b)**. Operating as above but using 3,4-(methylenedioxy)aniline (**12**) (0.480 g; 3.5 mmol) was obtained compound **9b**. Yield: 43%; m.p. 151.2-151.9 °C (white leaflets from ethyl acetate - petroleum ether). ^{1}H NMR (DMSO-d$_6$, 400 MHz): δ 10.04 (bs, 1H, NH); 7.61 (d, *J*=7.8 Hz, 1H, H-4); 7.42 (d, *J*=7.2 Hz, 1H, H-7); 7.39 (s, 1H, H-2); 7.33-7.19 (m, 6H, H-6, 2H-o, 2H-m, H-2'); 7.09 (t, *J*=7.2 Hz, 1H, H-p); 7.01 (t, *J*=7.4 Hz, 1H, H-5); 6.96 (d, *J*=8.4 Hz, 1H, H-6'); 6.83 (d, *J*=8.4 Hz, 1H, H-5'); 5.96 (s, 2H, OCH_2O); 5.38 (s, 2H, NCH_2); 3.70 (s, 2H, CH_2CO). ^{13}C NMR (DMSO-d$_6$, 100 MHz): δ 169.1; 147.0; 142.7; 138.3; 135.9; 133.8; 128.5; 127.8; 127.5; 127.3; 127.1; 121.2; 119.0; 118.7; 111.9; 110.0; 108.5; 108.0; 101.2; 100.8; 48.9; 33.4. ESI: *m/z* 385 (M + 1). HRMS: *m/z* calcd for $C_{24}H_{20}N_2O_3$: 384.1474; found: 385.1545.

Syntheses of compounds **9c**, **9d** and intermediate **15**

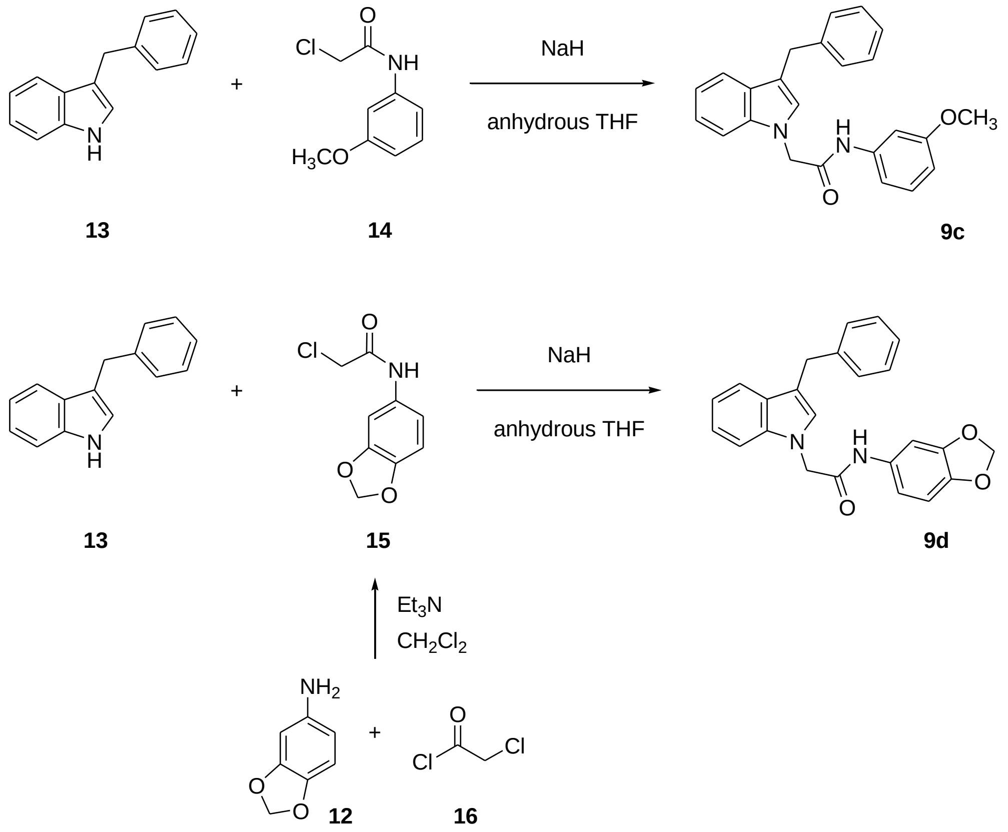

*2-(3-Benzyl-1*H*-indol-1-yl)-*N*-(3-methoxyphenyl)acetamide* **(9c)**. To anhydrous THF (1.5 mL), at 0 °C, sodium hydride (11.4 mmol) and then dropwise a solution of 3-benzylindole (**13**) (0.18 g; 1.03 mmol) in anhydrous THF (1 mL) were added. The mixture was stirred at 0 °C for 30 min and then the 2-chloro-*N*-(3-methoxyphenyl)acetamide (**14**) (0.23 g; 1.14 mmol) in anhydrous THF (1 mL) was added. The mixture was stirred at room temperature for 1 h (TLC analysis; ethyl acetate:petroleum ether = 1:2 v/v as eluent). The reaction mixture was added of methanol (5 mL) and poured in diethyl ether (30 mL) then deionized water was added (30 mL). The organic layer was separated and dried on Na_2SO_4. Removal of the solvent left a solid which was washed with cold diethyl ether to give a white solid. Yield 82%; m.p. 154.4-157.7 °C. ^{1}H NMR (DMSO-d$_6$, 400 MHz): δ 10.30 (bs, 1H, NH); 7.44 (d, *J*=7.8 Hz, 1H, H-4); 7.34 (d, *J*=8.2 Hz, 1H, H-7); 7.30-7.02 (m, 9H, H-6, Ph, H-2', H-5', H-6'); 6.96 (t, *J*=7.4 Hz, 1H, H-5); 6.62 (d, *J*=7.8 Hz, 1H, H-4'); 4.97 (s, 2H, CH_2CO); 4.03 (s, 2H, CH_2); 3.69 (s, 3H, Me). ^{13}C NMR (DMSO-d$_6$, 100 MHz): δ 166.5; 159.5; 141.4; 139.9; 136.8; 129.6; 128.4; 128.2; 127.7; 127.5; 125.7; 121.2; 118.7; 118.6; 113.6; 111.3; 109.6; 109.0; 104.8; 54.9; 49.0; 30.8. ESI: *m/z* 371 (M + 1). HRMS: *m/z* calcd for $C_{24}H_{23}N_2O_2$: 370.1681; found: 371.1747.

*N-1,3-benzodioxol-5-yl-2-(3-benzyl-1*H*-indol-1-yl)acetamide* **(9d)**. Operating as above but using chloro derivative **15** (0.24 g; 1.1 mmol) was obtained compound **9d**. Yield: 40%; m.p. 183.5-187.0 °C. ^{1}H NMR (DMSO-d$_6$, 400 MHz): δ 10.26 (bs, 1H, NH); 7.45 (d, *J*=7.0 Hz, 1H, H-4); 7.36 (d, *J*=8.6 Hz, 1H, H-7); 7.29-6.96 (m, 10H, H-2, H-5, H-6, Ph, H-2', H-6'); 6.86 (d, *J*=8.6 Hz, 1H, H-5'); 5.98 (s, 2H, OCH_2O); 4.95 (s, 2H, CH_2CO); 4.03 (s, 2H, CH_2). ^{13}C NMR (DMSO-d$_6$, 100 MHz): δ 166.0; 147.1;

143.0; 141.4; 136.8; 133.1; 128.5; 128.2; 127.7; 127.5; 125.7, 121.2; 118.8; 118.6; 113.6; 112.0; 109.7; 108.1; 101.2; 101.0; 48.9; 30.8.

N-*1,3-benzodioxol-5-yl-2-chloroacetamide* **(15)**. Chloroacetyl chloride (**16**) (1.03 mL; 1.47 g; 13 mmol) was added dropwise to a mixture of 3,4-(methylenedioxy)aniline (**12**) (1.65 g; 12 mmol) and triethylamine (2.8 mL; 20 mmol) in anhydrous CH_2Cl_2 (7 mL) at 0–5 °C. The reaction mixture was stirred at 0–5 °C for 30 min and at room temperature for a further 1 h. The progress of the reaction was monitored by thin layer chromatography using toluene:acetone = 4:1 v/v as eluent. The reaction mixture was then poured into crushed ice. The organic layer was separated, washed successively with $NaHCO_3$ solution and water, and dried over Na_2SO_4. Evaporation under reduced pressure gave a solid that, purified by flash-chromatography (ethyl acetate:petroleum ether = 1:2 v/v as eluent), gave the desired compound **15** (85%). ^{1}H NMR (DMSO-d_6, 400 MHz): δ 10.24 (bs, 1H, NH); 7.23 (d, *J*=1.6 Hz, 1H, H-2'); 6.93 (dd, *J*=8.7 Hz, *J*=1.6 Hz, 1H, H-6'); 6.85 (d, 1H *J*=8.7 Hz, H-5'); 5.96 (s, 2H, OCH_2O); 4.17 (s, 2H, CH_2CO). ^{13}C NMR (DMSO-d_6, 100 MHz): δ 164.7; 147.4; 143.7; 132.9; 112.8; 108.4; 101.9; 101.4; 43.7. ESI: *m/z* 385 (M + 1). HRMS: *m/z* calcd for $C_{24}H_{20}N_2O_3$: 384.1474; found: 385.1544.

Biology

Cell growth conditions. Human cervix carcinoma (HeLa) cells were grown in DMEM medium (Gibco, Milano, Italy), supplemented with 115 units/mL penicillin G (Gibco, Milano, Italy), 115 μg/mL streptomycin (Invitrogen, Milano, Italy), and 10% foetal bovine serum (Invitrogen, Milano, Italy).

Analysis of ARE-luciferase reporter gene activity. Hela cells were transiently transfected with ARE-luciferase reporter[4] using calcium phosphate transfection method. Cells were co-transfected with pMAX plasmid to normalize luciferase detection. After 24 h from transfection, the culture medium was replaced with fresh medium containing *t*-BHQ (Sigma-Aldrich, Milano, Italy) or the tested compound at the indicated concentration. Following treatment, cells were harvested and processed for analysis of luciferase activity according to manufacturer instruction (Dual-Luciferase Reporter Assay System; Promega). They were analyzed using a microplate luminometer (Victor3™; Perkin-Elmer, Waltham, MA, USA).

Western Blot Analysis. HeLa cells were incubated in the presence of the tested compound and, after 24 h, were collected, centrifuged, and washed two times with ice cold phosphate buffered saline (PBS). The pellet was then resuspended in lysis buffer. After the cells were lysed on ice for 30 min, lysates were centrifuged at 15000 x g at 4 °C for 10 min. The protein concentration in the supernatant was determined using the BCA protein assay reagents (Pierce, Italy). Equal amounts of protein (10 µg) were resolved using sodium dodecyl sulfate-polyacrylamide gel electrophoresis (SDS-PAGE) (Criterion Precast, BioRad, Italy) and transferred to a PVDF Hybond-P membrane (GE Healthcare). Membranes were blocked with a bovine serum albumin solution (5% in Tween PBS 1X), the membranes being gently rotated overnight at 4 °C. Membranes were then incubated with primary antibodies against Nrf2, NQO1, TKT (all from Santa Cruz Biotechnology), or β-actin (Sigma-Aldrich) for 2 h at room temperature. Membranes were next incubated with peroxidase labeled secondary antibodies for 60 min. All membranes were visualized using ECL Select (GE Healthcare),

and images were acquired using an Uvitec-Alliance imaging system (Uvitec, Cambridge, UK). To ensure equal protein loading, each membrane was stripped and reprobed with anti-β-actin antibody. The results of our experiments are reported in Figure S1.

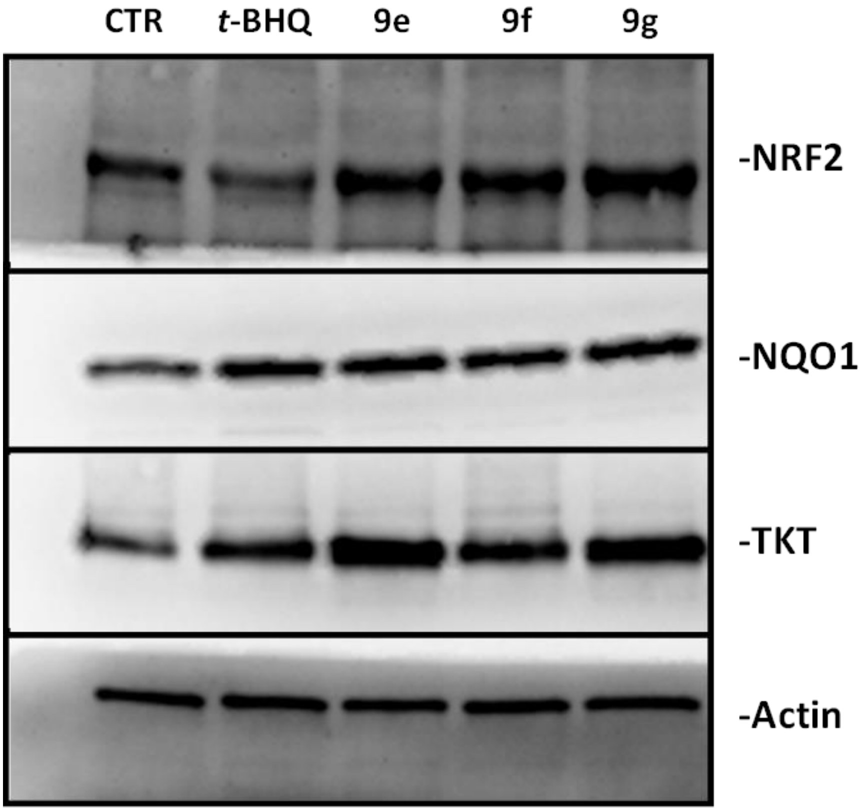

Figure S1 Western blot analysis of Nrf2, NQO1 and TKT after 24 h of treatment of HeLa cells with the indicated compounds. Compounds **9e** and **9f** were used at the concentration of 10 μM, whereas **9g** was used at the concentration of 5 μM due to its limited solubility in phosphate buffer. *t*-BHQ was used at the concentration of 50 μM as a positive control. To confirm equal protein loading, each membrane was stripped and reprobed with anti-β-actin antibody.

Evaluation of cytotoxicity of compounds ***9e-g***. Peripheral blood lymphocytes (PBLs) from healthy donors were obtained by separation on a Lymphoprep (Fresenius KABI Norge AS) gradient. After extensive washing, cells were resuspended (1.0 x 10^6 cells/mL) in RPMI-1640 with 10% fetal bovine serum and incubated overnight. For cytotoxicity evaluations in proliferating PBL cultures, non-adherent cells were resuspended at 5 x 10^5 cells/mL in growth medium, containing 2.5 μg/mL PHA (Irvine Scientific). Different concentrations of the tested compounds were added and viability was determined 72 h later by the MTT test as previously described.[5] For cytotoxicity evaluations in resting PBL cultures, non-adherent cells were resuspended (5 x 10^5 cells/mL) and treated for 72 h with the test compounds, as described above. The results of the cytotoxicity assays for compounds **9e**-**g** are reported in Table S1.

Table S1 Cytotoxicity of compounds **9e**-**g** for human peripheral blood lymphocytes (PBLs).

	GI_{50} (μM)[a]	
compound	$PBL_{resting}$[b]	PBL_{PHA}[c]
**9e**	> 100[d]	61,0 ± 12,9
**9f**	> 100[e]	84,9 ± 5,9
**9g**[f]	> 5[g]	> 5[h]

[a] Compound concentration required to inhibit cell growth by 50%. [b] not stimulated with PHA. [c] stimulated with PHA. [d] **9e** at the concentration of 100 μM killed 35% of the $PBL_{resting}$. [e] **9f** at the concentration of 100 μM killed 31% of the $PBL_{resting}$. [f] **9g** was tested at the concentration of 5 μM owing to its limited solubility in phosphate buffer. [g] **9g** at the concentration of 5 μM killed 6% of the $PBL_{resting}$. [g] **9g** at the concentration of 5 μM killed 10% of the PBL_{PHA}.

Computational chemistry

Design of indole derivatives assisted by molecular modeling. An in-house small library of indole derivatives was converted into 3D structures through the molecular modeling software suite Maestro.[6] The RCSB PDB website[7] was interrogated for Keap1 crystal structures in complex with a small molecule inhibitor of the Keap1-Nrf2 interaction. Using Maestro, the inhibitors were separated from the macromolecules. Then, our indole derivatives were screened through the Maestro Shape Screening tool using default settings and employing the co-crystallized ligands as templates. The best matching indole derivative/co-crystal inhibitor pair resulted between **9h** and the furane derivative labelled **FUU** (PDB code 3VNG).[8] Herein, we report examples of superimpositions of the molecular models of **9h** and **9g** on FUU (Figure S2).

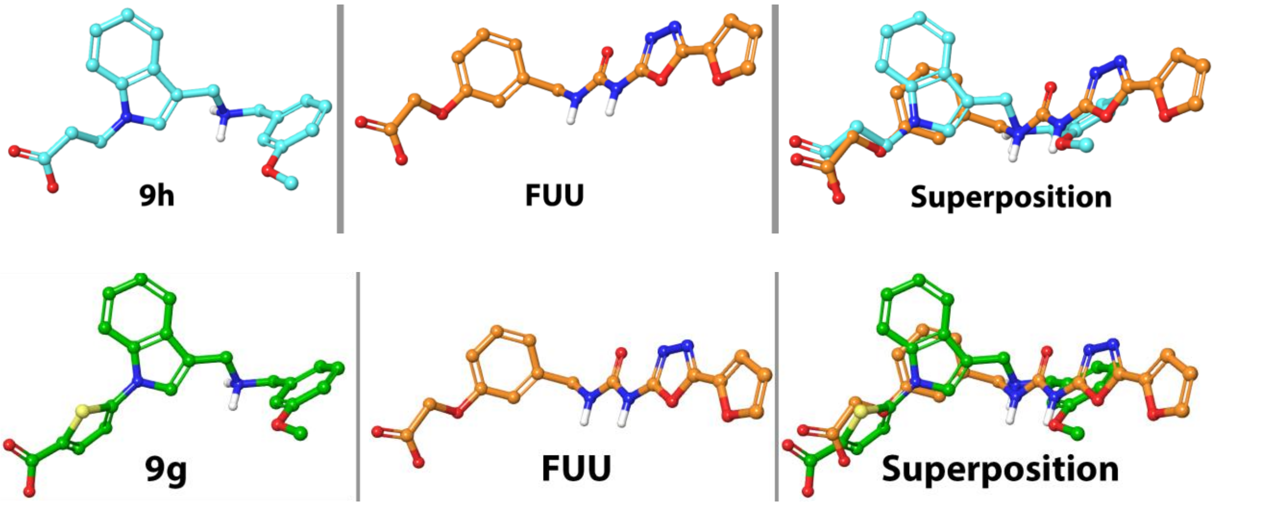

Figure S2 Superposition of **9h** (top) and **9g** (bottom) on FUU, a small molecule co-crystallized with Keap1 (PDB code 3VNG[8]).

Example of identification of commercially available indole derivatives using the SciFinder[9] substructure search routine. Taking **9h** as a reference structure, we searched similar commercially available indole derivatives bearing a tricyclic aromatic ring as a linker between the indole nitrogen and a carboxylic group. For such a purpose, we built up a query for substructure search in SciFinder and obtained three hits among which we selected **9g** (Figure S3).

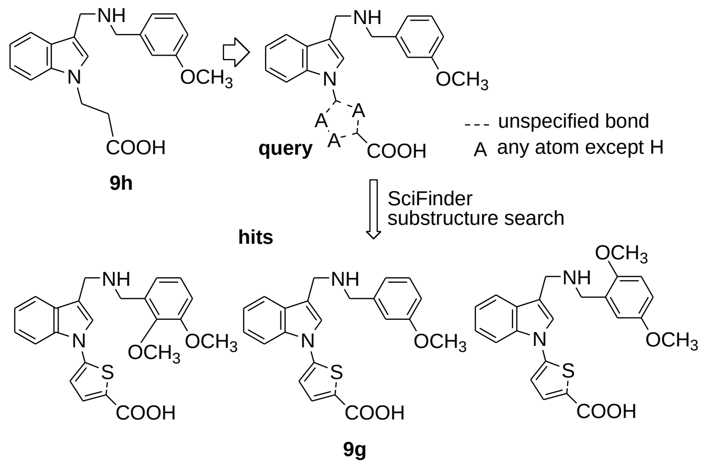

Figure S3 Example of our approach to find commercially available compounds using the Scifinder substructure search routine.

*Docking of compound **9g** into the 3D structure of Keap1 solved by X-ray diffractometry*. Compound **9g** was docked into all the available X-ray structures of Keap1 in which this protein was co-crystallized in complex with a low molecular weight binder. This ensemble-docking approach[10,11] was made necessary because Keap1 features a certain flexibility of the side chains of the residues lining the protein binding cavity. Thus, the X-ray complexes having PDB codes 3VNG,[8] 3WNK,[12] 4IFN,[10] 4IQK,[11] 4L7B, AL7D, 4N1B[12] 4L7D and 4XMB[13] were all employed in our docking calculations with the latest version of AutoDock4.2 (AD4) program.[14]

The proteins were prepared for the docking using the Protein Preparation Wizard of the Maestro suite[6] which assigns bond orders, adds hydrogen atoms, deletes water molecules and generates the appropriate protonation states. The co-crystal ligand of each complex was deleted from the cognate protein. The 2D Sketcher tool of Maestro was used to build **9g**. Of this ligand, the protonation and tautomeric state, and its geometry, were optimized through LigPrep, part of the same suite. Through Maestro, each prepared Keap1 structure was superimposed on the one corresponding to the PDB code 3WNK. Compound **9g** and the Keap1 protein structures were converted to the AD4 specific file format (PDBQT) using the python scripts prepare_ligand4.py and prepare_receptor4.py, part of AutoDock Tools (ADT) ADT by applying the default settings. The docking area was centered on the putative binding site. For each protein structure, a set of grids of 60 Å × 60 Å × 60 Å with 0.375 Å spacing was calculated around the docking area for the ligand atom types using AutoGrid4. For each ligand, 100 separate docking calculations were performed. Each docking calculation consisted of 10 million energy evaluations using the Lamarckian genetic algorithm local search (GALS) method. The GALS method evaluates a population of possible docking solutions and propagates the most successful individuals from each generation into the subsequent generation of possible solutions. A low-frequency local search according to the method of Solis and Wets[15] is applied to docking trials to ensure that the final solution represents

a local minimum. All dockings were performed with a population size of 250, and 300 rounds of Solis and Wets local search were applied with a probability of 0.06. A mutation rate of 0.02 and a crossover rate of 0.8 were used to generate new docking trials for subsequent generations, and the best individual from each generation was propagated over the next generation. The docking results from each of the 100 calculations were clustered on the basis of root-mean square deviation (rmsd) (solutions differing by less than 2.0 Å) between the Cartesian coordinates of the atoms and were ranked on the basis of free energy of binding (ΔG_{AD4}). We considered the docking solution having the lowest predicted binding free energy. According to this criterion, **9g** would establish the tightest binding interactions (ΔG_{AD4} -9.45 kcal/mol and frequency of occurrence 61/100) with the X-ray structure having PDB code 4L7B.

*Calculation of physicochemical and pharmacokinetic properties of **9a**-**i**.* Table **2** lists the physicochemical and pharmacokinetic properties of compounds **9a**-**i** calculated using the Maestro QikProp tool,[13] that predicts the widest variety of pharmaceutically relevant properties.

Table S2. Calculated Physicochemical and Pharmacokinetic Properties of Compounds **9a**-**i**.

Compound	9a	9b	9c	9d	9e	9f	9g	9h	9i	Range of recommended values[a]
#rotor[b]	6	5	6	5	5	6	6	8	9	0 – 15
#rtvFG[c]	0	1	0	1	0	0	0	0	0	0 – 2
Lipinski Rule of 5 violations[d]	1	1	1	1	1	1	0	0	0	N.A.
mol_MW[e]	370.45	384.43	370.45	384.43	378.44	408.471	392.47	338.40	352.43	130.0 – 725.0
dipole[f]	5.64	6.13	3.94	6.14	6.58	6.47	5.45	4.94	2.04	1.0 – 12.5
SASA[g]	687.44	644.38	673.71	659.67	672.61	699.77	707.092	641.14	656.52	300.0–1000.0
donorHB[h]	1	1	1	1	2	2	2	2	2	0.0 – 6.0
accptHB[i]	3.25	4	3.25	4	3.75	4.5	4.25	4.25	4.25	2.0 – 20.0
QPlogPo/w[j]	5.89	5.23	5.83	5.37	5.11	5.16	2.91	2.16	2.27	-2.0 – 6.5
QPlogS[k]	-6.64	-5.74	-6.39	-6.01	-6.32	-6.40	-6.03	-4.42	-4.52	–6.5 – 0.5
QPPCaco[l]	4805.82	4348.64	4646.53	4698.04	177.45	187.31	48.78	75.16	49.00	< 25 poor, > 500 great
Jorgensen Rule of 3 violations[m]	1	1	1	1	1	2	1	0	0	N.A.
#metab[n]	4	2	4	2	6	7	5	5	5	1 – 8
Human Oral Absorption[o]	100%	100%	100%	100%	84.19%	84.90%	74.18%	73.15%	70.51%	< 25% poor, > 80% high

[a]For 95% of known drugs. [b]Number of non-trivial (not CX3), non-hindered (not alkene, amide, small ring) rotatable bonds. [c]Number of reactive functional groups. [d]Predicted numbers of violations of Lipinski's rule of five. [e] Molecular weight of the molecule (Da). [f]Computed dipole moment of the molecule (debye). [g]Total solvent accessible surface area (SASA) in square angstroms using a probe with a 1.4 Å radius. [h]Estimated number of hydrogen bonds that would be donated by the solute to water molecules in an aqueous solution. [i]Estimated number of hydrogen bonds that would be accepted by the solute from water molecules in an aqueous solution. [j]Predicted *n*-octanol/water partition coefficient. [k]Predicted aqueous

solubility, log S. S in mol dm^{-3} is the concentration of the solute in a saturated solution that is in equilibrium with the crystalline solid. [l]Predicted apparent Caco-2 cell permeability in nm/sec. Caco-2 cells are a model for the gut-blood barrier. [m]Predicted numbers of violations of Jorgensen rule of three. [n]Number of likely metabolic reactions. [o]Predicted qualitative human oral absorption percentage.

References

1) Xu Q, Huang L, et al. Design, synthesis and biological evaluation of thiazole- and indole-based derivatives for the treatment of type II diabetes. Eur J Med Chem 2012;52:70–81.
2) De Rosa M, Soriente A. Rapid and general protocol towards catalyst-free Friedel–Crafts C-alkylation of indoles in water assisted by microwave irradiation. Eur J Org Chem 2010:1029–32.
3) Modh RP, Prasanth Kumar S, et al. Design, synthesis, biological evaluation, and molecular modeling of coumarin–piperazine derivatives as acetylcholinesterase inhibitors. Arch Pharm Chem Life Sci 2013;346:793–804.
4) Wang XJ, Hayes JD, Wolf CR. Generation of a stable antioxidant response element–driven reporter gene cell line and its use to show redox-dependent activation of Nrf2 by cancer chemotherapeutic agents. Cancer Res 2006;66:10983–94.
5) Romagnoli R, Baraldi PG, et al. Concise synthesis and biological evaluation of 2-aroyl-5-amino Benzo[*b*]thiophene derivatives as a novel class of potent antimitotic agents. J Med Chem 2013;56, 9296-9309
6) Schrödinger Release 2018**:** Maestro, Schrödinger, LLC, New York, NY, 2018.
7) https://www.rcsb.org
8) Satoh M, Saburi H, et al. Multiple binding modes of a small molecule to human Keap1 revealed by X-ray crystallography and molecular dynamics simulation. FEBS Open Bio 2015;5:557-70, https://www.rcsb.org/structure/3vng
9) Gabrielson, SW. SciFinder, J Med Libr Assoc 2018;106:588–90.
10) Cosconati S, Forli S, et al. Virtual screening with AutoDock: Theory and practice. Expert Opin Drug Discov 2010;5:597-607.
11) Cosconati S, Marinelli L, et al. Protein flexibility in virtual screening: The BACE-1 case study. J Chem Inf Model 2012;52:2697–2704.
12) Suzuki N, Fujimoto, et al. Structural elucidation of the cyclization mechanism of α-1,6-glucan by *Bacillus circulans* T-3040 cycloisomaltooligosaccharide glucanotransferase. J Biol Chem 2014;289:12040–51.
10) Crystal Structures of apo Keap1, Keap1-peptide, and Keap1-compound complexes, https://www.rcsb.org/structure/4ifn
11) Marcotte, D, Zeng W, et al. Small molecules inhibit the interaction of Nrf2 and the Keap1 Kelch domain through a non-covalent mechanism. Bioorg Med Chem 2013;21:4011−19.
12) Jnoff E, Albrecht C, et al. Binding mode and structure-activity relationships around direct inhibitors of the Nrf2-Keap1 complex. ChemMedComm 2014;9:699–705.
13) Jain AD, Potteti H, et al. Probing the structural requirements of non-electrophilic naphthalene-based Nrf2 activators. Eur J Med Chem 2015;103:252–68.
14) Morris GM, Huey R, et al. AutoDock4 and AutoDockTools4: Automated docking with selective receptor flexibility. J Comput Chem 2009;30:2785–91.
15) Solis FJ, Wets RJ-B. Minimization by random search techniques. Math Oper Res 1981;6:19-30.
